# Development and innovation in a new distributed medical programme: Scottish Graduate Entry Medicine (ScotGEM)

**DOI:** 10.3389/fmed.2025.1586851

**Published:** 2025-07-14

**Authors:** Fiona Graham, Jon Dowell, Angela Flynn, Shalini Gupta, Andrew David MacFarlane, Andrew O’Malley, Robert Scully, Francis Michael Sullivan, Lloyd Samuel John Thompson, Kirsty Alexander

**Affiliations:** ^1^School of Medicine, University of Dundee, Dundee, United Kingdom; ^2^School of Medicine, University of St Andrews, St Andrews, United Kingdom; ^3^NHS Education for Scotland, Edinburgh, United Kingdom; ^4^School of Medicine, University of Galway, Galway, Ireland

**Keywords:** distributed training, undergraduate medical education, rural and remote areas, Scotland, family medicine and general practice, social accountability

## Abstract

**Introduction:**

Addressing the shortage of primary-care physicians, especially in remote and rural areas, is a crucial target in many countries. This article introduces the Scottish Graduate Entry Medicine (ScotGEM) programme: a compressed, tailor-made curriculum designed to equip and enthuse its graduates to practice generalist and rural medicine in Scotland, within the ethos of socially accountable medicine.

**Methods:**

This curriculum paper describes ScotGEM in sufficient detail for the reader to translate elements to their own context. It then collates findings from evaluations, research projects and many critical discussions about the programme. This work is used to describe and evaluate the curriculum design and delivery, with a focus on the distributed aspects.

**Results:**

Three key innovations of the curriculum are explored in detail: the Generalist Clinical Mentor (GCM) role; the year-long primary care Longitudinal Integrated Clerkship (LIC); and the Agents of Change curriculum. There are early signs that ScotGEM is encouraging generalist, rural careers within Scotland. There is also growing evidence of the benefits ScotGEM faculty and students bring to the clinical workforce in the distributed settings.

**Discussion:**

Distributed programmes require additional organization for students and faculty. Partnerships can be challenging but immensely rewarding. Healthcare partners in rural areas need to be involved early in planning and strong relationships fostered with local “champions.”

## 1 Introduction: background and rationale for the educational activity innovation

Addressing the shortage of primary-care physicians is a growing concern in many countries (1). In Scotland, United Kingdom, these shortages amount to a crisis (2). Recruitment into the specialty of General Practice (Family Medicine) is below sustainable levels (3, 4). Poor retention is also a key issue, with many General Practitioners (GP) taking early retirement or leaving the profession (5). Shortages are particularly stark in rural areas, where primary care is often very heavily relied upon (6). Increasing the GP workforce in Scotland across underserved areas is fundamental to the functioning of the state-funded National Health Service (NHS) and thus to maintaining the health of the nation.

Addressing workforce shortages, and the factors causing these, is central to Scottish health and social care policy (7). This response was strongly informed by two key reports that provided guidance and strategies for medical schools to encourage and support their graduates into general practice, including increasing the amount of GP teaching to undergraduate medical students (8, 9). In parallel, the government increased the number of medical places in primary-care focused courses in Scotland by 22% over 5 years (10).

Beyond activities to increase and retain the workforce, there has been a drive to make healthcare more compassionate, safe and sustainable for patients and staff. This is spearheaded in Scotland by the initiative of Realistic Medicine, introduced in 2014–15 (11), now in its eighth version “Taking Care” (12). This resonates with international initiatives such as “Prudent Healthcare” in Wales (13) “Slow Medicine Italy” (14) and “Choosing Wisely” in the United States (15). A foundation of this approach is developing a generalist medical workforce (16).

As part of all these directives, a Scottish Government initiative was launched in 2016 to create a new medical programme focused on educating students for generalist, rural practice in Scotland, who would also contribute to socially accountable and sustainable practice (17). From this, the Scottish Graduate Entry Medicine Programme (ScotGEM) was created and accepted its first intake in 2018 with the stated mission:

*To produce top quality, adaptable, compassionate, generalist doctors who will help drive change in the delivery of healthcare across Scotland.*

Scottish Graduate Entry Medicine is a distributed programme with students and faculty studying and working across four partner Scottish Health Boards (NHS Highland in the north, NHS Fife and NHS Tayside centrally, and NHS Dumfries and Galloway in the south) as part of a programme led by the University of St Andrews and the University of Dundee. It is also Scotland’s first graduate-entry only medical programme and offers a compressed, tailor-made curriculum designed to equip and enthuse its graduates to practice generalist and rural medicine in Scotland, within the ethos of socially accountable and realistic medicine.

This article describes ScotGEM’s novel curriculum to offer insight into how it utilizes distributed training innovations to achieve these aims, and to share practical considerations. In doing so, we aim to contribute understanding about how innovative curricula for distributed programmes may be developed and implemented for the benefit of students, clinical practices, workforce distribution and rural communities.

## 2 Pedagogical framework(s), pedagogical principles, competencies/standards underlying the educational activity

**FIGURE 1:**
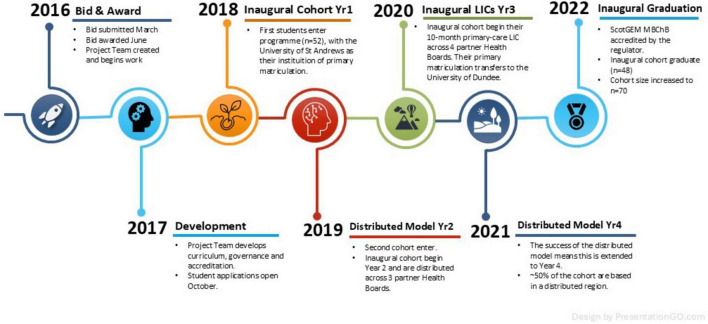
Timeline of ScotGEM development and inaugural cohort.

The ScotGEM proposal was developed in response to the Scottish government’s call for bids to provide a new 4-year graduate medical programme for Scotland. It was finalized over approximately 6 months, submitted to competitive tender in March 2016, and awarded in June 2016. Over the following 2 years, the detail of the curriculum was developed at unusual speed by a project team led by the two medical schools and in conjunction with the four partner NHS Health Boards and the University of the Highlands and Islands (UHI). At the Scottish Government’s request, and with the support of the United Kingdom Medical Regulator the General Medical Council (GMC), standard approvals processes were expedited to accredit the course. Applications opened to students in October 2017, with the first cohort (*n* = 52) entering in September 2018 (Figure 1).

Curriculum design and approval, as well as advertising and recruitment, were done under immense time pressure. Thus, drawing on existing expertise from external policies and existing curricula, as well as at the founding partner institutions, was essential to develop the cornerstones of the novel curriculum. For example, the community-led and rurally based educational approach drew heavily on recommendations within the aforementioned “*By Choice Not Chance”* report (8). Visits to learn about the University of Melbourne’s and Flinders University’s rural community based Longitudinal Integrated Clerkship (LIC) provided insight into how these ran in established settings and convinced the team to make this an integral part of the ScotGEM bid. Valuable learning from delivering these in the local context was also gained from the general practice curriculum at the University of Dundee, which already included an optional year-long rural LIC for a small number of students. This LIC was evaluated via staff and student feedback (18) as well as a published qualitative study using interviews and focus groups with students (*n* = 7), health service staff (*n* = 4), GP tutors (*n* = 21) and reflective audio-diaries kept by all students (19). Later, a key supportive relationship was established with Prof Roger Strasser of Northern Ontario School of Medicine when it transpired that their well-established curriculum that had several strong similarities to ScotGEM (20, 21).

It was decided that the curriculum’s underlying pedagogy should be constructivist, aiming to build students’ learning based upon their existing knowledge, experiences in the course and reflection. A review of the literature and visits to the University of Warwick and University of Southampton graduate entry courses helped to select and develop Case Based Learning (CBL) as the best approach in the first 2 years. This was mapped into a framework of intended learning outcomes (ILOs) (22), which drew extensively on the GMC’s required Outcomes for Graduates (23, 24) as well as all the United Kingdom medical Royal Colleges undergraduate learning outcomes lists [see, e.g., (25)]. This led to the development of an extensive 3D matrix from which 54 CBL scenarios and other activities were identified, with ILOs distributed systematically across them.

The original ScotGEM proposal presented a distributed approach in two key regards. First, with two universities offering a joint programme in which students enjoyed joint matriculation (and access to facilities) throughout, but primary matriculation lay with St Andrews in years one and two, Dundee in years three and four. This was key as it determined what regulations applied when. Secondly, the bid included partnerships with four geographically dispersed largely rural NHS Health Boards and a proposed full year GP LIC. This was well received by the Scottish Government. Although complex to establish, the resulting collaboration was also strongly supported by the NHS Education Scotland funding allocation process and promised to generate fresh enthusiasm and thus capacity for undergraduate teaching within these diverse locations.

The proposal submitted to the Scottish government bid for 40 student places on ScotGEM, and 55 were awarded when the bid was accepted. This increased again to 70 places in 2022. It was decided to accept graduates of any discipline, as the emerging evidence base supported this (26), leading to an admissions process that utilized two distinct components. Academic aptitude, including a scientific knowledge base, was assessed by the Graduate Medical School Admissions Test (GAMSAT). This was followed by a 10 station Multiple Mini Interview (MMI) that, in part, favored those seeking a generalist focused, distributed course and mature applicants. For an analysis of Dundee Medical School’s experience of MMIs, see Dowell et al. (27).

For a new course in a highly regulated profession, appropriate standard setting and quality assurance was a key focus. For ScotGEM, this was facilitated by access to the two parent Medical Schools’ examination question banks and assessment systems, including the existing progress test operated by the University of Dundee Medical School. This enabled ScotGEM to operate annual stage appropriate summative exams and offer a yearly formative assessment to illustrate the standard required by the end of the programme to students. Once ScotGEM students reached the end of their third (penultimate) year it was possible to make direct performance comparisons with the fourth-year students on the standard-entry University of Dundee course (also completing their penultimate year). This is a key milestone of the course, when students are required to sit the Applied Knowledge Test (AKT) of the GMC Medical Licensing Assessment (28).

The regulatory burden on ScotGEM’s development was significant. As a new programme developed under the aegis of two universities, it was subject to the review and approval of both, as well as the standard GMC regulatory processes and the NHS Education Scotland quality assurance procedures. These processes were onerous, but reassured stakeholders that the intended and actual educational delivery was appropriate and of an acceptable standard. Additional external guidance was provided by Prof Val Wass, Professor of Medical Education in Primary Care at University of Aberdeen, and Dr Paul Garrud, founding Director of the Derby Graduate Entry Medicine programme. They also engaged in a mock quality assurance panel in advance of the formal processes.

## 3 Distributed learning environment (setting, students, faculty); learning objectives; pedagogical format

The Distributed Learning environment for ScotGEM is important, not just for high quality learning, but to support the mission to develop generalist, rural clinicians through appropriate and sustained clinical exposure to these settings and the clinicians within them (29, 30). This ethos led to the development of the Generalist Clinical Mentor (GCM) role and the Agents of Change curriculum, which are embedded within all years of the course (described below). Students also complete a portfolio across the 4 years with the support of one consistent faculty member as a supervisor, complementing ScotGEM’s emphasis on longitudinal learning. The portfolio plays a key role in supporting students to develop as professionals and demonstrate professionalism.

Clinical exposure takes place from the first week of the course. In the context of CBL, a bespoke Clinical Interactions Course (CLIC) was developed to teach clinical and communication skills across the first 2 years of the programme. This is delivered on a weekly basis, largely by GCMs (see below) who then take their small group of (*n* = 6–8) students into their own practice to apply and develop their knowledge in a real-world setting. In Year 1, weekly CLIC teaching is based in the Clinical Skills Center in the University of St Andrews. From Year 2, the students are dispersed across Scotland rotating between three of the four partner Health Boards: NHS Highland in the north, NHS Fife centrally, and NHS Dumfries and Galloway in the south. This exposes students to the myriad of settings where healthcare is delivered as well as to different approaches to learning (e.g., small rural Health Boards do not have purpose built Clinical Skills suites but have embraced the opportunity to develop effective learning using existing environments, including study and social spaces for students, often shared with postgraduate medical and other healthcare learners). Students report that the consistent and high volume of patient interaction helps to prepare them for their time as residents, and that exposure to the patient journey in a range of settings provides a more holistic view of patients, as reflected in this excerpt from an independent Visit Report by the United Kingdom medical regulator (GMC) on ScotGEM 2018–19.

*In particular, students value the contact they have with patients from the very beginning of the course and told us that they feel comfortable interacting with real patients already (31).*

A learning point for the delivery team was the appreciation of the additional communication, organization and logistics that distributed programmes require, and the importance of shared decision-making and open communication channels. Clear communication with applicants and entrants is also important: students need to be prepared for the implications of a distributed programme, including financial (see e.g., an independent student opinion piece (32)). To support with finances, all students are offered a bursary, and many are eligible for a fee reduction or waiver (33). Particularly during the initial years, whilst university and regional staff were getting used to the new curriculum, the feedback from students and clinicians was essential to refine the fit of processes and policies. For example, see this excerpt from an independent Visit Report by the GMC on ScotGEM 2020–21.

*Year two students told us that the clinical exposure they had so far had been fantastic. They stated that they were still going into practices to meet with their GCMs and they felt this experience was valuable. Students told us some placements felt slightly disorganized, with staff not knowing the students were supposed to be there when they arrived, they have fed this back to the schools and things improved (34).*

**FIGURE 2:**
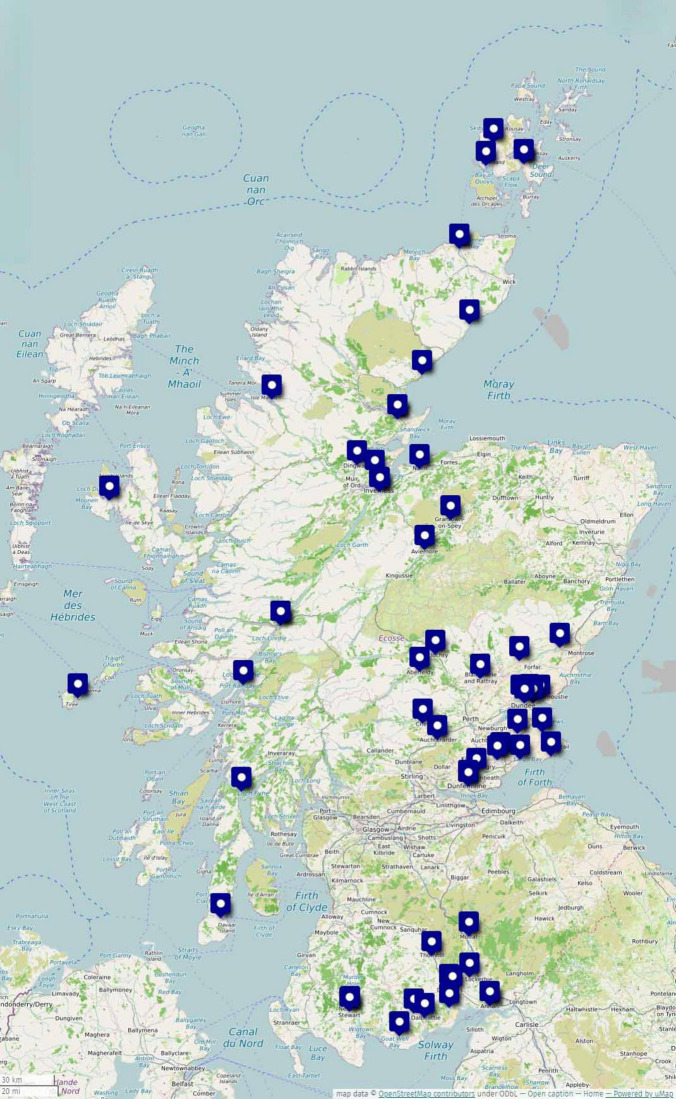
Distribution of ScotGEM LIC practices 2023–24.

Except for their University of Dundee induction week, Year 3 students are based in an assigned general practice for the academic year (10 months) during their LIC. Integration into the practice environment and team is essential to successful learning and thriving, particularly in remote and rural settings. Students are dispersed across the country from the Orkney Islands in the north, to the south-west corner of Scotland (see Figure 2). The LIC experience is explored in detail below.

In the final year (Year 4), ScotGEM students follow the same curriculum as the students in the final year (Year 5) of the MBChB course at the University of Dundee. Although it was originally envisaged that all final year students would be based at the School of Medicine in Dundee, the success of the distributed model in the earlier years led to students requesting, and being offered, the opportunity to spend their final year in one of the other partner Health Boards. Around 50% of each cohort have chosen this option to date. The differences between the experience of students in the distributed settings and those centrally include:

1. Smaller groups in distributed settings can facilitate clinical experience, knowledge of individual students and possibly individualized support.
2. Preferences for clinical placements (Students Selected Components or SSCs) may be more effectively tailored to learning needs in distributed settings, because of the smaller numbers.
3. Centrally located students have a teaching hospital experience unlike the distributed students. All students have one 4-week Acute Care Block in a teaching hospital to help address this potential inequality.
4. Centrally located students can “benchmark” against their “equivalent” University of Dundee students. All settings may offer a similar opportunity in relation to students from other Scottish programmes.

## 4 Results to date/assessment (processes and tools; data planned or already gathered)

With an original curriculum, as well as clear mission and political drivers underpinning the establishment of ScotGEM, evaluation of the course is imperative. In this section, we consider the development of three key innovations through which ScotGEM’s mission has been operationalized: the novel GCM role; a year-long primary care LIC; and the Agents of Change curriculum. We also report on the initial workplace impacts of the programme – careers intentions and decisions for the first cohorts, and the benefits ScotGEM faculty bring to the clinical workforce in the distributed settings.

We employed a dialogical approach to explore the development and impact of ScotGEM, and to build the claims presented in this paper. This involved regular interactions between the authors where we discussed and critiqued aspects of the course, collated evidence, and decided which elements to include. Within this process we were mindful of making assumptions, given that almost all authors are “insiders” to the programme. FG, JD, AO’M, RS, LT, FS were deeply involved in the initial development and set up of ScotGEM, and FG, JD, AF, AO’M, RS, LT, KA contribute, or have contributed to, its current delivery and development. AMacF provides the perspective of a ScotGEM student from the inaugural cohort, who graduated in 2022 and is now in postgraduate training. Although this intimate knowledge of the programme permitted more nuanced understandings and analysis, we also acknowledge that our experiences inevitably influence our interpretations and that an (unconscious) desire to present facts in a certain light might be at play. Thus, to mitigate potential bias, we practiced a reflexive approach. This involved regular discussions to critique others’ claims and critical reading of each other’s written contributions. SG provided an external view, as she is involved in medical education at the University of Dundee, but not within ScotGEM. We also required that citable evidence was provided to support claims and could ideally be triangulated. This evidence comes from a variety of sources including published studies, independent regulatory reports, internal evaluations and informal feedback. When referencing this evidence, we have included brief details in the text to allow readers to judge its likely reliability and scope.

### 4.1 The generalist clinical mentor (GCM)

The GCM role, conceived for the ScotGEM programme, involves employing clinical generalists in a multi-dimensional clinical-academic role providing mentorship, teaching and clinical services within university and affiliated clinical teaching practice settings (35). This aims to leverage the positive impact that longitudinal mentorship (36) and role-modeling can have on students’ eventual career decisions (37), whilst also helping make the programme socially accountable by supporting the local clinical services, which are often in underserved locations.

General Practitioners were targeted for the role as they exemplify community-embedded generalists, able to provide an authentic learning experience that aligned with the case-based curriculum (9). GCMs are supported to provide this multi-dimensional role, through enhanced induction, and continuous professional development opportunities. GCMs were recruited through open competition (interview, with Health Board and University representatives on the selection board), with the option of University, NHS or partnership contracts. This flexibility was felt to be important, but most applicants elected for NHS contracts due to pension considerations. Criteria for the role were broad, but all GCMs had to be established, independent clinicians, to enable effective embedding within host clinical sites. Formal medical education experience and qualifications was desirable, but not essential, recognizing the importance the programme placed on mentorship and role-modeling, which could be demonstrated through various means. To mitigate against the variable backgrounds of candidates, an 8-week induction programme was arranged for all GCMs, as well as fortnightly educational meetings (with hybrid options for colleagues in rural locations), an annual quality review meeting (in-person, with social and networking opportunities), generous CPD opportunities, and a half-day per week for discretionary development work. At steady state, forty-one GCMs were employed across the four partner Health Boards, all on less-than-full-time contracts. Retention in the role has been high, with formal and informal feedback crediting combined clinical academic contracts as the principle reason for this. A potential detractor from the sustainability of the role was an initial lack of progression relating to academic rank, however, some GCMs have now been appointed to medical education leadership roles e.g., in clinical skills and within partner Health Boards.

Students learn with their GCMs in small groups (4–8 students) throughout all years of the course, however, the group size, frequency, contact-time and duration of the sessions vary (see Table 1). Small groups provide students with significant time with their GCM (up to 40% of academic contact time in Years 1 and 2). Drawing on a master-apprentice model, GCMs provide weekly practice-based clinical contextualization during the compressed CBL medical curriculum (38).

**TABLE 1:** Generalist Clinical Mentor (GCM) group size, frequency and duration per academic year.

Cohort	GCM group size	Frequency of small group	Duration of small group	Duration of academic year
Year 1	6–8	Weekly, for 12 h over 2 days	15 weeks	30 weeks
Year 2	4–8	Weekly, for 12 h over 2 days	6–18 weeks	36 weeks
Year 3	6–8	Weekly, for 4 h	40 weeks	40 weeks
Year 4	6–8	Monthly, for 4 h	40 weeks	42 weeks

In contrast to the literature on goal-orientated group mentorship, the ScotGEM GCMs are embedded within the programme and have a longitudinal interaction with small groups of mentees, but with a general rather than a specific goal (36). The aim of this style of general mentorship is to provide longitudinal role-modeling in multiple settings (e.g., clinical skills laboratory, clinical practice, tutorials), whilst also developing a meaningful and trusted relationship with the mentee. The GCM model has received positive feedback since the inception of the course, and remains critical to the successful delivery of the programme. For example, see this excerpt from an independent Visit Report by the GMC on ScotGEM 2018–19.

*Generalist Clinical Mentors are a valuable part of the programme and students were very positive about their experience of learning with them (31).*

The GCM role changed as per the requirements of the programme. In Year 1 and 2, GCMs deliver clinical, communication and procedural skills teaching to small groups, whilst also contextualizing this learning with real patients each week in their host clinical environment. In Year 3, the GCMs provide weekly small group teaching, facilitation and support for students on longitudinal integrated clerkships. In Year 4, the GCMs provide monthly small group half-day release, with a focus on professionalism, preparedness for practice, and generalism (see Table 1 for frequency and duration of the small groups).

Throughout all years, the GCMs provide informal support to their students, as well as delivering multiple other functions including recruitment, assessment, career-advice and supervision, scholarship, research and programme evaluation.

### 4.2 The longitudinal integrated clerkship (LIC)

The LIC was integral to the ScotGEM bid, given the potential of LICs to aid the recruitment and retention of a sustainable workforce (39) and the increasing evidence that LICs provide high-quality education, build students’ generalist skills and develop their sense of social accountability (40, 41). The continuity of care and long-term relationships with patients and mentors, and prolonged exposure to the challenges and rewards of living and working in these settings, are key factors contributing to these positive outcomes (42, 43). The ScotGEM LIC is a year-long primary-care placement, designed to fulfill the criteria of a “comprehensive LIC” (44), and includes practices in Scotland’s most remote and rural areas (see Figure 2). The LIC in its current form began as a pilot project within the standard-entry University of Dundee Medicine Programme (18). The University and NHS teams worked closely together to identify and recruit local GP practices who could take part in the LIC model. With the advent of ScotGEM, the number of students in the LIC has increased from 8 students at the height of the pilot in 2018, to around 75 students in 2025. To support this significant increase in numbers, partner Health Board-based clinicians have been appointed to lead on practice recruitment, working closely and collaboratively with the university team. This local knowledge base and contact is crucial to the identification of suitable practices and to the processes of engagement and support in distributed settings.

Building and maintaining strong relationships with practices has been vital to the success of the LIC. Initial data from internal evaluation shows a high GP practice retention rate with an average of 85% of practices continuing to take students year on year. Practices particularly value the mentorship aspect of having a LIC student for the year, as well as positive patient experiences of being involved in medical education. As students develop their competence and skill over the LIC year, many practices feel that their LIC student positively contributes to clinical workload capacity as the year progresses. ScotGEM faculty undertake regular quality assurance visits to GP practices to identify areas of good practice and support any areas needing development. These visits form part of the wider, regular, interaction with GP tutors and practices, which includes in-person and online tutor development activities to build and maintain ongoing relationships.

The presence of LIC students in GP practices can also have a positive impact on patient and clinician experience. Evidence from a phenomenological, cross-sectional qualitative study involving semi-structured interviews with patients (*n* = 5) who had experienced several contacts with LIC students via the Dundee LIC pilot, described how LIC students can empower the patient during consultations (45). The first cohort of ScotGEM students undertook their LICs during the height of the Covid-19 pandemic, which posed unprecedented new challenges with social distancing, significant changes to consultations and staff redeployment. A research study of the experiences of ScotGEM GP tutors during this turbulent time provides important lessons for the organization and delivery of LICs (see (46), an inductive thematic analysis of interview data with *n* = 8 GP tutors across varied practices in Scotland). In keeping with the published literature, [see e.g., (47)] feedback from ScotGEM LIC practices report students becoming part of the practice team, leading to increased motivation among staff and an enthusiasm on teaching and learning [McElhinney et al., in preparation^1^, a qualitative research study drawing on interview data with LIC GP Tutors (*n* = 8)]. The inclusive, supportive relationship between preceptors and students also reassured educators of the efficacy of the LIC for students during early external quality assurance of the programme. For example, see this excerpt from an independent Visit Report by GMC on ScotGEM 2021–22.

*Year three students spoke very highly of the support they have received on placements during their third year. Students told us that whilst they were initially nervous of the transition to the third year, they felt incredibly supported in the transition. Students praised the support they received from the clinical teams whilst on placement, for both primary and secondary care (48).*

The year, however, has required refinement. By design, LICs allow “*white space*” to allow students to independently direct their learning and focus on their own learning priorities and goals (49). When designing the course, the project team followed evidence that this flexible and self-directed structure would the style of graduate-entrants well (50). Subsequently only 60% of the 40-hour work-week is timetabled, with students arranging their own secondary care placements, portfolio work and self-study in the remaining 40%. A published thematic analysis of interview data on how ScotGEM students (*n* = 13) utilized their “*white space*” (51) revealed that although some students thrived on the opportunity to personalize their learning and juggle other commitments, others required more direction to effectively organize and make use of learning opportunities. Subsequently, the dedicated resource for support for students in the LIC year has been increased, whilst the amount of portfolio assessment during this year has been reduced. The intent is to allow students more time to use their “*white space*” effectively. We also plan to provide more direct organization of secondary care learning experiences, ensuring that students in each setting undertakes a broadly similar number and range of hospital-based learning experiences.

### 4.3 Agents of Change

ScotGEM’s underlying social accountability ethos led to the inclusion of an Agents of Change (AoC) strand of the curriculum. This aims to support students to develop the skills, knowledge and mindsets to drive positive change in diverse healthcare systems throughout their medical careers. AoC is spiral curriculum that revisits five key themes with increasing complexity: service Learning; Healthcare Informatics; Quality Improvement (QI); Prescribing and Therapeutics; and Public Health (see Table 2). By embedding these elements into medical training, ScotGEM aims for students to have the competence and confidence to become active agents of change in their medical careers.

**TABLE 2:** Key themes and formats of assessment within Agents of Change.

Key theme	Focus of student learning	Format of assessment (Year of students)
Service learning	To engage directly with the communities where they are based to develop a deeper understanding of community engagement and the social determinants of health, including access to care, health literacy, and environmental factors.	Year 1: Service-Learning placements and projects Year 3: Community engagement projects
Healthcare informatics	To learn how to harness data analytics, electronic health records, and other technologies to support clinical decision making and with whole systems thinking approaches.	Year 1: Prescribing audit Year 2: Critical Appraisal Journal clubs
Quality improvement	To develop the skills and mindset to identify and implement continuous improvement in a range of healthcare settings.	Year 2: Group quality Improvement project Year 3: Individual healthcare improvement projects; Significant event analysis Year 4: Healthcare improvement projects; Adverse event review
Prescribing and therapeutics	To develop the skills and knowledge to reduce medication errors and improve therapeutic outcomes.	Year 1: Prescribing project Year 2: Quality Improvement project Year 3: Healthcare improvement projects; Significant event analysis Year 4: Healthcare improvement projects; Adverse event review
Public health	To enable decision making based on evidence, patient circumstances, and clinical guidelines to address population-level health challenges	Year 1: Prescribing audit Year 2: Critical Appraisal Journal clubs Year 3: Healthcare improvement project Year 4: Healthcare improvement project

The distributed nature of the programme provides a unique advantage in engaging healthcare providers and communities. Conducting AoC projects within real-world environments (e.g., GP practices, hospital wards, community settings), offers students the benefits of experiential learning (52): valuable insights into the realities of healthcare delivery across and beyond traditional clinical environments, and the varied challenges faced by healthcare systems and communities. For example, from the first semester of

Year 1, students are introduced to the concept of Service Learning (see Table 1). During the LIC year, when students are distributed across Scotland (see Figure 2), students are required to engage with the community in which they are based through a service-learning project which supports development of an understanding of the social determinants of health and a holistic approach to patient care (53). Students can follow their own interests and local health or social needs to choose a project and how to engage. ScotGEM students have run lifeboat stations, volunteered in youth groups, community fridges and LGBTQ + groups, supported community initiatives from cinemas to wild swimming and mentored aspiring doctors. The aim of these activities is to position students as role models and support essential services, helping revitalize these areas whilst they develop as holistic professionals. Working on projects with diverse patient populations may help students gain varied medical experience about individual patient care but also learn to tailor this care to meet the unique needs of various communities and improve community health outcomes. An ongoing mapping process facilitates a continuous and agile assessment of how the curriculum and its implementation meets its educational goals and adapts to the evolving healthcare landscape.

In line with the principles of Realistic Medicine (12), the competencies taught through AoC are vital to ensure that future doctors can lead efforts to reduce medication errors, improve therapeutic outcomes and deliver quality, value-based medicine. QI principles are embedded throughout the curriculum to help students develop a mindset focused on continuous improvement (see Table 1). QI project initiatives encourage students to design and implement projects that are practical, meaningful and responsive to the complexities of modern healthcare. In their LIC year, ScotGEM students are supported to undertake a mandatory, substantial healthcare improvement project whilst in their practice, which can also lead to evidenced improvements in clinical practice and patient care [see e.g., two published studies reporting findings from these projects (54, 55)].

### 4.4 Workforce impact

How close is ScotGEM to achieving its aims? In the United Kingdom, doctors must undertake 4–6 years of undergraduate education, 2 years of Foundation training and at least 3 years of postgraduate training before they are considered fully qualified specialists or GPs. Additionally, in the United Kingdom, a third of doctors plan to leave the NHS after completing their Foundation or postgraduate training (56). This further increases the duration between graduation and completion of specialty/GP training. Individuals from the first graduating cohort of ScotGEM, who have not experienced any delays or breaks, are only now eligible for specialty/GP training. Consequently, available data remains limited. However, early indications indicate significant positive impact in relation to the primary objectives of the ScotGEM programme.

Data from recent ScotGEM student surveys of career intentions indicate that 83% of graduates aim to remain in Scotland, 80% plan to pursue a career in General Practice, and 50% intend to work in rural or remote areas (57). These surveys are sent routinely throughout students’ time on the course to capture intentions longitudinally. Although the data also show students are commonly still undecided about their career, overall, 89% intend to pursue a career aligned with one of the programme’s three key performance indicators, which are to either remain in Scotland, work as a clinical generalist, or practice in remote/rural environments.

Initial graduate destination data collected by the United Kingdom Foundation Programme (UKFPO) similarly supports these trends: so far, each year 87%–90% of ScotGEM graduates remain in Scotland for the subsequent year for Foundation Training. In comparison, a retrospective cohort study (58) indicates 73% (613/841) of Scottish medical schools’ graduates remain in Scotland at this point. Unpublished data suggests that these trends continue, with data from 2024 showing 90% of ScotGEM graduates remained to work in Scotland, in comparison to 74% from all Scottish Schools. Furthermore, among graduates from the inaugural cohort who were contactable 2 years after completing foundation training (*n* = 27; response rate 64%), 90% continued their training in Scotland. 44.4% “strongly agreed” or “agreed” that living and working in a rural area was important to them *(we thank the UKFPO, personal communication, 2024, for sharing this data)*.

Longer term follow-up using the United Kingdom Medical Education Database (UKMED) (59) will enable us to better understand the patterns and complexities of ScotGEM students’ career intentions and destinations.

One aim of ScotGEM, to drive change in the delivery of healthcare in Scotland, extends beyond positive outcomes for the students. The distributed nature of ScotGEM has supported the development of academic clinicians in remote and rural areas across primary and secondary care which is a policy goal (60). The development and appointment of GCMs as the lynchpin of student learning has led to a new cohort of educators in the partner Health Boards, who are simultaneously supporting the clinical workforce. For example, in NHS Fife (serves a population of 370, 000, with no major urban center), the GCM model currently provides an additional 4.2 full-time equivalent of GP service into the health system (across 20 GP practices) alongside additional contributions to secondary and unscheduled care.

As of 2025, the LIC engages 78 medical educators as GP tutors in 57 GP practices across Scotland. Scores of primary and secondary care clinicians have become involved in ScotGEM as block leads, specialty leads, Student Selected Component supervisors, portfolio supervisors, professionalism tutors and QI Project supervisors. Additionally, many more clinicians in rural areas have become involved in teaching, examinations and admissions, and are planning, or undertaking, professional development such as qualifications in medical education.

## 5 Discussion on the practical implications, objectives and lessons learned

The innovative ScotGEM programme is proving successful in producing flexible, compassionate, high-quality graduates. There are early signs of its effectiveness in encouraging generalist, rural careers within Scotland. For a programme grounded in the principles of social accountability, we are also proud that ScotGEM’s distributed model positively impacts on rural communities beyond the aim of increasing rural doctors.

Distributed medical programmes require “more” of their students in relation to additional organization, travel, accommodation and adapting to a large variety of learning environments. Regular communication and feedback between students and faculty in the Health Boards and the universities has been imperative in refining the curriculum, distinguishing roles, embedding points of contact and supporting students. In Table 3, a recent ScotGEM graduate reflects on the student experience.

**TABLE 3:** A graduate perspective.

The geographical spread of the course is key in expanding the career horizon of students who live, work, learn and explore different regions of Scotland. It removes a strong negative factor in choosing rural generalism embedded in the traditional medical education course design – the unknown of working and living in a rural setting in Scotland. ScotGEM students know what living in a rural setting looks like, and the opportunities and benefits it can afford to their professional and personal life. Moreover, in the early years, students form communities of practice with their peers and are supported by a GCM who can act as a powerful positive role model in developing an interest in rural generalism. Although many ScotGEM students and recent graduates may still be undecided about their eventual career path, it may be that the choice to live and work rurally can be made with a confidence that graduates from other courses lack.

Another success of the distributed model is the fostering of clinical educators in “non-traditional” settings, in this case rural Scotland. In the past a medical education or academic career necessitated living near a medical school, usually in a city. The GCM initiative demonstrates that clinical academics not only teach and nurture future doctors but can also support the local clinical service more immediately. This has relevance for partnerships between clinical authorities and higher education institutions. ScotGEM has appointed educators in rural areas to substantive roles but has also facilitated involvement in medical education for a far greater number of clinicians in areas such as selection, examination and mentorship as well as teaching. At a time when medical careers are increasingly demanding, and recruitment and retention is at critical levels, we might postulate that this initiative has an important role to play in increasing job satisfaction and preventing burnout. With respect to the challenges of the programme, two major factors emerged. The first was the relative expense of the programme compared to more traditional models. Students are dispersed around the country from Year 2 thus travel and accommodation costs are high. In Year 3, when many are based in rural areas, this has had a significant fiscal impact. Students also must contend with a higher cost of living, limited accommodation and longer distances to travel, for example, between their practice and the secondary care placement provider. These expenses may represent the biggest practical risk to the programme in the longer term although it has remained in surplus since its inception. The second factor has been the small number of students who professed an interest in joining a generalist, remote and rurally focused course at interview but were subsequently unhappy about rural placements. Whilst most subsequently had successful placements, it was disappointing that the admission processes were sometimes unable to identify authentic motivation. This has led to a review of the admissions processes, including consideration of interviews being based in rural areas and delivered by rural practitioners, to emphasize the importance of the distributed model and the programme mission, and active efforts to attract applicants from remote and rural areas.

Like Scotland, other countries are actively looking to mission-led medical education programmes to address shortages in primary care and rural physicians. When establishing ScotGEM, we drew on the experiences of other programmes (see section “2. Pedagogical framework(s), pedagogical principles, competencies/standards underlying the educational activity”). Now, when comparing the challenges and success of the programme to that of others, our findings suggest that aspects are translatable to other contexts. For example, introducing a distributed elements to a programme can bring additional clinicians to rural communities, as well as develop the existing workforce [see e.g., (53, 61)]; and building active community engagement and healthcare improvement into the curriculum can be challenging but allows understanding and mutual benefit [see e.g., (62–64)].

Key lessons from ScotGEM for other settings:

1. Distributed programmes require faculty to pay particular attention to relationship building, communication and additional organization, travel, accommodation and learning environments.
2. Partnerships can be challenging (e.g., regulation, communication) but bring significant rewards when the strengths of all organizations are harnessed.
3. Healthcare providers in rural areas (in this case Health Boards) need to be involved early in planning distributed programmes and strong relationships fostered with local “champions.” Local leadership is essential to key programme areas e.g., recruitment of teaching staff and LIC practices.
4. Educator involvement in a wide variety of activities, e.g., portfolio supervision, examination, block leadership etc., helps foster a sense of belonging in a distributed programme and allows rural clinicians to tailor their involvement to the time available.
5. Ambitious initiatives such as the Agents of Change Programme take time to embed. Clarity of aims are important as well as dissemination of successful student projects.

## 6 Acknowledgment of any conceptual, methodological, environmental, or material constraints

As a “Curriculum, Instruction, and Pedagogy” contribution, this paper does not adhere to the traditional scientific format of introduction, methods, results and discussion. Rather, it collates a summary of evaluations, research projects and many discussions conducted over time. It aims to describe and evaluate the curriculum design and delivery, with a focus on the distributed aspects. This article references research and evaluation undertaken on the ScotGEM programme. Many studies were authored by ScotGEM staff and conducted with the rigor required to be published as original research. Others are primarily attributable to ScotGEM students. However, we also draw on Visit Reports by the GMC which were conducted independently of ScotGEM staff. We have striven for a reflexive, balanced approach in the article, using strong quality evidence where this exists. However, we acknowledge that additional independent assessment of ScotGEM would be beneficial. We highlight ongoing challenges to the programme in this paper and prioritize its ongoing evaluation.

Given the wide scope of this descriptive paper, we have not reported the methodological detail for each study individually in the text, rather have signposted readers to the publication type and methodology. Readers who wish to assess rigor and/or see further detail to enable them to replicate included aspects in their own context can do so in the following ways: (1) The majority of studies are published, cited accordingly and can be accessed in the published domain; (2) ongoing research and evaluation that is currently in preparation or under consideration and is not yet publicly available may be obtained from the authors via request to the corresponding author.

## Data Availability

The data analyzed in this study is subject to the following licenses/restrictions. Restrictions for institutional evaluative data. Requests to access these datasets should be directed to FGraham001@dundee.ac.uk.

## Footnotes

1 McElhinney Z, Alexander K, Hoskison G, Barlett M. 2025. It makes you up your game, and so it should: The impact of hosting a LIC student on GP tutors clinical practice. School of Medicine, University of Dundee.
